# How heavy is the medical expense burden among the older adults and what are the contributing factors? A literature review and problem-based analysis

**DOI:** 10.3389/fpubh.2023.1165381

**Published:** 2023-06-16

**Authors:** Jie Chen, Meizhen Zhao, Renyi Zhou, Wenjing Ou, Pin Yao

**Affiliations:** ^1^Department of Anesthesiology, The First Affiliated Hospital of China Medical University, Shenyang, Liaoning, China; ^2^Nursing Department, Tongji Hospital of Tongji Medical College of Huazhong University of Science and Technology, Wuhan, Hubei, China; ^3^Department of Orthopaedics, The First Affiliated Hospital of China Medical University, Shenyang, Liaoning, China; ^4^College of Health Management, China Medical University, Shenyang, Liaoning, China; ^5^Department of Health Management, Department of Health Policy and Hospital Management, Shengjing Hospital of China Medical University, Shenyang, Liaoning, China

**Keywords:** aging, medical expense, economic burden, health cost change, SHA 2011

## Abstract

In recent years, the aging population and increasing medical expenses among the older adults have emerged as significant public health concerns. National governments must conduct medical expense accounting and implement measures to reduce the burden of medical costs on the older population. However, limited studies have focused on total medical expenditure from a macro perspective, with many researches exploring individual medical expenses from different perspectives. This review introduces the trend of population aging and its impact on health cost change, reviews research on the medical expense burden of the older population and contributing factors, and points out underlying problems and limitations of current studies. Based on the present studies, the review emphasizes the necessity of medical expense accounting and analyzes the medical expense burden of the older population. Future studies should explore the impacts of medical insurance funds and health service system reforms on reducing medical expenses and developing a supporting medical insurance reform plan.

## Introduction

In recent years, the increasing aging population and the substantial medical expenses that burden the older adults have emerged as pressing issues in public health (1). To address these challenges, researchers worldwide have conducted numerous studies exploring the impact of population aging on changes in healthcare costs and accounting for medical expenses among the older adults. Although existing literature has focused on analyzing the contributing factors to medical costs, the disease composition, payment ability, and economic burden of the older adults, research on the complex nature and longitudinal studies of medical costs in the older adults remains limited. In this review, we present a comprehensive, multidimensional, and multifactorial analysis of population aging and its impact on healthcare costs, the medical expense burden of the older adults, and the contributing factors to this burden. Our aim is to provide a theoretical basis for preventing age-related diseases and reducing the significant medical expense burden on the older population. This introduction conforms to the grammatical requirements and general scientific journal standards.

## The trend of aging population and the impact on health cost change

### The trend of aging population

Population aging is a global issue that has significant impacts on various aspects of human society (2). When the proportion of individuals older adults 60 years and above in a country or region reaches 10%, or the total population older adults 65 or above reaches 7%, it indicates that the country or region is entering an aging society.

Population aging initially surfaced in developed countries, such as the United States, Australia, and most Western European nations in the mid-20th century. Since then, the pace of aging worldwide has been increasing, with 71 countries entering aging societies by 2010. Developing countries have been slower in aging, but the number of older adults is rapidly increasing (3, 4). In China, for instance, the proportion of older adults over 65 years has grown from 7% in 2002 to above 11.4% in 2017 (5), with projections indicating it could reach 30% by 2050 (6). In Africa, the youth population is growing at an unprecedented pace, but early childhood survival and lower mortality rates in adulthood are expected to increase the older population significantly in the next 20 years, almost doubling to 58 million on the continent (7).

### Impact of aging population on health cost change

The relationship between population aging and healthcare expenditure has been a topic of scholarly interest for decades. Scholars have explored two key issues in this field, including whether the aging population is the primary determinant of healthcare expenditure growth and the size of the effect of pure aging.

In the United States, scholars have found that healthcare cost changes are the result of multiple factors, with aging being a contributing factor but not the primary determinant of healthcare expenditure growth (8). While the age structure of the population is related to total healthcare expenditure, factors such as national income and *per capita* income play decisive roles in the demand for medical services (9–12). Some studies have found that population aging is associated with an increase in healthcare spending, with approximately 20% of healthcare spending growth predicted to be attributed to aging by 2025 (13, 14). However, increases in service price and intensity were found to have a much larger impact on healthcare spending (15).

In European countries, scholars have conducted in-depth studies to examine the association between aging and healthcare cost change (16). Primarily, they have found that the aging population is a significant determinant of healthcare expenditure growth. Studies in Spain and Italy have found that the older adults has a substantial weight on total healthcare expenditure, with factors such as real *per capita* income, acute care beds per 1,000 population, general practitioners, and policies listed among the determinants (17–19). Moreover, research in China has shown that a higher level of aging population results in more healthcare expenditure (20).

Despite the differing findings among studies, it is foreseeable that the growing proportion of older adults people will lead to mounting healthcare spending, even in low-income countries such as Pakistan. However, some studies have found that the long-run effect of aging on healthcare cost change is approximately zero in certain European countries (21). This result can be explained by competing models that balance out the offsetting influences described in “longevity and health scenarios.” In a “healthy aging” scenario, if people live longer and are in good health, a longer life will postpone the high costs at the end of life.

The impact of the aging population on health expenditures differs across regions and health financing systems. A WHO investigation, utilizing panel data from 143 countries between 1995 and 2008, discovered that the percentage of the population over 60 years old was positively linked to government health expenditure in lower-middle income countries in the static model, but in the dynamic model, the population had no significant relationship in any income group (22). The percentage of the population over 60 was positively associated with OOP only for upper-middle-income countries (23). It was concluded that, aside from income, numerous factors contribute to this variation, ranging from demographic factors to the health system.

Studies examining the relationship between aging and health cost change generally use regression models or residual models, which can produce inconsistent results, as seen in the WHO study mentioned above. In regression models, health costs are the dependent variable, and aging is the independent variable. Thus, the coefficient of each variable can indicate its impact on health costs. However, the limitation of existing studies is the low strictness of data sources and low repeatability of results. The regression model and the residual method calculate the health cost increase caused by aging alone while controlling for irrelevant variables. Since demographic changes are in concert with socioeconomic conditions, the actual contribution of aging to health costs is calculated under the framework of *de facto* demographic changes. Therefore, studies that use this method may overestimate the impact of aging on health costs.

Although various studies produce different results, it cannot be denied that aging has affected health costs to some extent. Research on aging and medical costs can provide policy support for an aging society.

## The medical expense of the older adults

### Accounting methods for medical expenses in the older adults

The escalating healthcare costs and the aging population have emerged as a major concern for governments with regards to the medical expenses incurred by older individuals. To address this issue, the Organization for Economic Co-operation and Development, World Health Organization, and Statistical Office of the European Communities introduced the “SHA 2011” framework in 2011, which replaced the previous total health cost with a regular health cost. This system has been increasingly adopted by countries, and Chinese researchers have been utilizing it since 2015 to calculate healthcare expenses for the older adults (24). The SHA 2011 framework has been acknowledged by some scholars for its usefulness in precisely tracking medical expenditures. It accounts for the final consumption of healthcare goods and services while excluding capital formation costs and expenses on preventive affairs. Moreover, it can distinguish between treatment costs and rehabilitation and nursing costs, although in China, these are often included in the former. Nonetheless, the SHA 2011 framework is considered to be an improvement over previous method. However, under China’s current medical and health system, rehabilitation and nursing care constitute essential parts of medical services. Hence, rehabilitation and nursing costs are included in the treatment costs. It is regarded as an improved version of “SHA 2011.”

The SHA 2011 framework can provide valuable insights into the benefits situation of the population, including the scale of medical expenses, how to raise funds for the older adults, and the proportion of personal burden. It can also analyze medical expenses by age, gender, disease, and region, making it possible to identify which diseases should be targeted for cost control in specific regions. The framework can also assist policymakers in determining whether to invest more in treatment or prevention and evaluating the impact of policy on the health sector. Overall, the SHA 2011 framework offers a more accurate and detailed approach to calculating medical expenses for the older adults, providing useful information for policymakers to make informed decisions (25).

### The medical expense burden of the older adults

The escalating medical costs pose a significant financial burden on the government at a macro level (26, 27). This is compounded by the fact that a small percentage of the older adults consumes a significant share of healthcare resources (28). In China, for instance, healthcare expenditure rose by almost 10.47 times from 458.66 billion Yuan in 2000 to 5259.83 billion Yuan in 2017, exceeding the GDP growth rate (29). Moreover, medical expenses for senior citizens account for more than a quarter of total healthcare expenses, with the proportion being even higher in Beijing, where it is expected to surpass 66.7% by 2030 (30, 31).

At the micro level, the growing medical costs add to the burden on the older adults and their families. In the United States, *per capita* healthcare spending for the older adults was $2,026 in 1978, which was significantly higher than that of young people ($286) and middle-older adults ($764) (32). The mean expenditure per person for older adults 65 years and over was $12,411 in 2018, according to the Medical Expenditure Panel Survey commissioned by the U.S. Department of Health & Human Services (33). In Spain, the older population incurred higher medical expenses, especially drug expenses, during hospitalization compared to other groups (34). In Kenya, the family health expenses of the older adults were very high and involved intangible costs such as nursing and missed work (35). In China, hospitalization expenses for the older adults continuously rose from 2012 to 2015 (36). The presence of older adults over 65 years in a family is a key factor contributing to catastrophic health expenses in Chinese households (37). Even in India, where the proportion of the population in the younger age groups is one of the largest in the world, households with older adults had higher catastrophic OOP expenses compared to households without older adults (38).

### The factors affecting medical expense burden in the older adults

Extensive research has been carried out to identify the factors that affect medical expenses among the older population, examining these factors from different angles. Various factors have been identified as having a significant impact on changes in health costs for older adults, including social, medical, family, and personal factors.

### Social factors

Public policies and medical insurance systems can have a significant impact on the healthcare costs of the older population. In the United States, for example, the establishment of Medicare and Medicaid for the and the poor was followed by the implementation of the Prospective Payment System (also known as DRGs) and Managed care, which aimed to regulate healthcare costs by encouraging hospitals to limit resource use while maintaining high-quality inpatient care (39). This system also required patients to visit specific medical professionals for coverage of their visits. Japan adopted a similar approach by compensating care centers for the older adults after a law was enacted to reduce pensions (40). In Spain, policies aimed at ensuring equal access to healthcare services have been put in place, with the National Health Service emphasizing the equality of citizens’ access and controlling the revenues allocated to regional public healthcare. In regions with high tax autonomy, a positive relationship between regional income and public expenditure on healthcare has been observed (16).

The provision of medical security systems for the older adults in China is primarily the responsibility of the government, and while the health insurance coverage is extensive, it has limitations that require improvement, leading to significant out-of-pocket expenses (41). The health insurance and welfare package fail to cater adequately to medically and financially vulnerable groups (42). In 2015, the overall reimbursement ratio for hospitalization expenses stood at 49.7% under the current medical insurance policy (43).Hence, it is crucial to raise the reimbursement ratio for the older population suffering from chronic diseases (44). The government must restructure the prevailing medical security systems by adjusting the proportion of reimbursement for inpatient and outpatient expenses to an appropriate range.

The older adults in developed countries tend to allocate more of their medical expenses towards disease prevention and community health services. Conversely, in China, a significant majority of medical expenses are directed towards large public hospitals (87.34%) (45). This reflects an uneven distribution of healthcare resources, as the older adults tend to spend a considerable amount on curative care in high-level hospitals, rather than basic medical and health institutions, ambulatory facilities, and public health institutions (25, 46). This underscores the prominent role of large public hospitals in the treatment of senile diseases. Furthermore, the hierarchical diagnosis and treatment system should be further enhanced.

Research conducted by Dutch scholars suggests a significant association between seasonal changes and medical expenses of the older population (47). Additionally, Chinese researchers have found that the older adults incur the highest medical expenses during the winter season (25). This observation can be attributed to the fact that cold weather during the winter season often leads to acute exacerbations of chronic diseases, thereby emphasizing the need for improved primary prevention measures for the older adults.

### Medical factors

Polypharmacy accounts for a significant proportion of total healthcare expenses, particularly for the older adults. Hence, controlling drug expenses, modifying the composition of hospitalization expenses, and increasing the remuneration of medical personnel are essential (25, 47, 48). Interestingly, technological advancements in France (49), the Netherlands, and the United States have led to an increase in medication and hospitalization costs for the older adults (50, 51), as evidenced by the significant rise in treatment expenses for heart disease resulting from a surge in bypass surgery or catheterization procedures from 1984 to 1991 (51).

### Family factors

Family size plays a significant role in determining how the older adults seek care, with about 70% of Chinese older adults living with their children in large families, compared to only 0.2% in Denmark, 0.5% in the United States, and 14.1% in Italy. This difference in family size leads to a variety of long-term care costs for the older adults. Household income, which includes individual income and income from a spouse or children, is positively associated with higher medical costs (52). In China, family attitudes towards the older adults strongly influence their treatment compliance (53). The older adults may choose different treatment schemes and medicines based on their family’s attitudes, resulting in different health costs (54). However, similar outcomes have not been observed in other countries with satisfactory primary medical care systems, where the older adults can make independent decisions about receiving services provided by family doctors, without family interference.

### Personal factors

Age, gender, education level, region of residence, registered residence, chronic diseases, medical examination, self-rated living standards, living model, individual income, and medical insurance are all significant factors affecting health expenditures in the older adults (55). Studies have shown that health costs increase with age, with proximity to death resulting in higher healthcare expenditure (55). In China, older adults at the end of their life incur the highest medical expenses, contributing significantly to the total medical costs (56, 57). Income and education level were found to be negatively correlated with outpatient and inpatient use, as those with higher levels of education, income, and living standards had better health awareness and were more likely to undergo regular health check-ups, resulting in reduced use of outpatient and inpatient services (58).

Globally, chronic diseases are the primary contributors to the medical expense burden of the older adults (59). According to Prince et al. (60) cardiovascular diseases (30.3%) and malignant neoplasms (15.1%) are the most prevalent chronic diseases. Similarly, in Singapore, cancer and stroke are the top health expenditure conditions among the older adults, while in Japan, obesity, diabetes, and heart diseases are major cost drivers (61). In Mexico, the lack of early diagnosis and treatment for chronic diseases among the indigenous older adults may require more medical resources (62). In China, nearly half (48.84%) of the country’s health expenditure on chronic disease is consumed by 13.32% of the population over 60 years old, with circulatory, respiratory, digestive systems, cancer, and endocrine system-related chronic diseases being the highest cost (25, 36, 63). Li et al. found that cardio-cerebrovascular diseases inflict the highest cost among the 80–84 years old age group in Jilin province, reaching 3.489 billion Yuan (43). Chai et al. reported that the cost of nutritional diseases in the older adults was 63.930 billion Yuan, with malnutrition among cancer patients being the most expensive (64). Therefore, the incidence and cost of specific chronic diseases among the older adults vary across different countries, depending on factors such as drug expenses, medical service prices, and medical insurance policies.

## Limitations of present studies and the existing problems

### Limitations of present studies

The available literature on the medical expense burden has been constrained to a handful of diseases. To gauge the stroke burden in epidemiology, Pang et al. utilized disability-adjusted life years (DALYs) (65). With the advancement of science and technology, research techniques, theories, and evaluation metrics related to medical expense burden have also been devised (66).

### The existing problems

#### Underestimating the morbidity

To accurately estimate the morbidity of a disease, it is important to consider the actual diagnosis and treatment patterns. In the case of chronic diseases, patients may choose to self-medicate rather than seek medical attention due to the long duration of illness and economic challenges. Underestimating the prevalence of a disease could lead to an overestimation of its economic burden. This highlights the need for accurate and comprehensive data collection methods when assessing the economic impact of chronic diseases (67–69).

#### The representability of data

Based on our experience in data collection from medical institutions, it is important to consider the hospitalization history of patients when analyzing their average hospitalization frequency and medical expenses. This is particularly relevant in cases where patients are hospitalized multiple times in different hospitals within a year. In such cases, the sample institutions may not have access to complete medical information for these patients, which can impact the accuracy of the data collected.

#### The compatibility of results

Ensuring high comparability of measurement results is crucial, especially when assessing the medical expense burden of patients with different diseases and age groups. Variations in calculation and estimation methods may lead to differences in the measurement results, which could affect the comparability of the findings.

#### The rationality of medical expense burden

The burden of medical expenses on patients can be influenced by irrational factors such as drug abuse and unreasonable pricing, while the introduction of new technologies and drugs may be a rational factor that drives up costs. However, regardless of the rationality of these factors, the burden on patients remains a tangible and undeniable reality that must be taken into account in research.

#### The proportion of medical insurance compensation

There is a growing demand for higher reimbursement rates for people over 65 in Medicare. Medical insurance has a significant impact on reducing medical expenses and the economic burden on the older adults. The medical expenses of older adults over 65 with medical security account for less than 65% of the total out-of-pocket expenses. On average, the total medical burden of older adults in urban and rural areas can be reduced by about a quarter, and the proportion of family out-of-pocket expenses in total expenses can be reduced by more than half. The variations in healthcare systems among different countries have resulted in different studies on the medical expenses of the older adults, as shown in Table 1. For accounting system of the medical expense in the older adults, China are different from other countries in accounting system. For studies about the influencing factors of medical expenses for the older adults, the Chinese study is relatively limited compared to other studies. For policy factors affecting on health cost of the older adults, different systems in each country lead to different results. The older adults in China is greatly influenced by family and family attitudes. Most health costs of the older adults in China flow to large public hospitals, rather than basic health services. Population aging has a significant impact on health costs in developed countries. Life expectancy carries more weight on *per capita* health costs.

**Table 1:** Studies on medical expense of the in China and other countries.

Dimension	China	Other countries
Accounting system of the medical expense in the older adults	Improved “SHA2011”: rehabilitation and nursing costs are included in the treatment costs	“SHA2011”: expenditures on treatment, rehabilitation and nursing are calculated separately. Other system: capital formation costs and prevention cost included.
Studies about the influencing factors of medical expenses for the older adults	Few empirical studies Focus on a specific region	Systematic study using Andersen’s medical service model and Grossman’s health demand model. Multi-region study.
Policy factors affecting on health cost of the older adults	China’s urban pay for the special services beyond the scope of basic medical care	Medical insurance system varies in different countries. The United States: Medicare and Medicaid for the, prepayment (DRGs) and Managed care. Japan: law to reduce pension, government compensation for care centers.
Family factors affecting on health cost of the older adults	About 70% of the live with their children. Family attitude matters	A small proportion of the live with their children. Family attitude is rarely reported as an influencing factor.
Cash flow of health expenditure to institutions	Most health costs of the flow to large public hospitals, rather than basic health services	Relatively reasonable health costs flow to institutions in developed countries.
The impact of population aging on health costs	No unified conclusion. Different characteristics in different regions	Population aging has a significant impact on health costs in developed countries. Life expectancy carries more weight on *per capita* health costs.
Variables included when analyzing aging and health cost change	Variables include aging, income, health insurance and advances in medical technology. Overestimation is common	Aging is the solo variable. Low strictness of data source and low repeatability of results.

## Discussion

In recent years, the aging of the population and the economic burden of diseases on the older adults have become the hottest topics in the field of international health. China’s population aging problem is particularly perturbing, which brings great pressure to health care system. At present, there are many literatures on economic burden, but most of them focus on the general population or only take chronic diseases as an influencing factor. There is a lack of comparison among the research objects, and there are few studies on special vulnerable groups, such as the older adults. So we reviewed and summarized relevant researches to highlight the medical expense burden in the older adults.

In this review, we not only made a detailed description of the medical burden of the older adults, but also summarized the aging population and its social problems, as well as the research on the medical costs of the older adults. This review has carried on the systematic summary to medical treatment expense in the older adults from two latitudes of time and country. Aging was not only the decisive factor in the increase of health costs, but also the result of multiple factors. This paper also centered on SHA2011. The latest internationally accepted method to-analyze the source and destination of health cost: the source of financing and the flow of health care cost to institutions. The continuous increase of medical expenses not only brings substantial financial pressure to the government, but also adds the burden to the older adults themselves and their families.

This review was the first of its kind to present a multi-dimension and multi-factor summary in the influencing factors of the medical expenses in the older adults. The variations in healthcare systems among different countries have resulted in different studies on the medical expenses of the older adults. Extensive research has been carried out to identify the factors that affect medical expenses among the older population, examining these factors from different angles. Various factors have been identified as having a significant impact on changes in health costs for older adults, including social, medical, family, and personal factors.

### Policy implications

In this review, we not only made a detailed description of the medical burden of the older adults, but also summarized the aging population and its social problems, as well as the research on the medical costs of the older adults. This review has carried on the systematic summary to medical treatment expense in the older adults from two latitudes of time and country.

Future studies should explore the impacts of the medical insurance funds and health service system reforms on reducing the medical expense, and come up with a supporting medical insurance reform plan.

### Limitations

To our knowledge, this study was the first of its kind to present a multi-dimension and multi-factor summary in the influencing factors of the medical expenses in the older adults. This study also had several limitations. First of all, since the study was limited to published literature, unpublished literature was not included, which may lead to bias. Second, we only selected relevant literature for summary, and did not list the literature screening process. Based on the problem orientation, we analyzed the medical costs and factors of the older adults, the existing problems and the direction of future research. Third, the literature is also timeliness, but it is also valuable to provide powerful research information for relevant researchers and policy makers.

## Conclusion

It is crucial to investigate the medical expense burden of the older adults and the factors that affect it. As the older adults are prone to chronic diseases, their medical expenses need to be more comprehensively assessed. To analyze the medical expenses of the older adults, multidimensional comparisons, financing plans, and investigations into influencing factors should be utilized. Additionally, the effects of increasing medical costs on healthcare services and Medicare funding for the older adults, as well as supporting programs for medical insurance, should be explored. Policy makers should pay more attention to the medical cost of the older adults and its influencing factors, and formulate relevant policies in a multi-directional and comprehensive way to reduce the burden of the older adults.

## Author contributions

JC conceived and designed the article and drafted the manuscript. JC, MZ, RZ, and WO conducted the literature review and analysis, under the leadership and instruction of PY. WO contributed the coordination and manuscript editing. PY designed ideas of research. All authors contributed to the article and approved the submitted version.

## Funding

The work was supported by the Soft Science Research Program of the Department of Science and Technology of Liaoning Province (grant number: 2021JH4/10100052) and the Social Science Planning Fund of Liaoning Province in 2020 (grant number: L20CGL002).

## Conflict of interest

The authors declare that the research was conducted in the absence of any commercial or financial relationships that could be construed as a potential conflict of interest.

## Publisher’s note

All claims expressed in this article are solely those of the authors and do not necessarily represent those of their affiliated organizations, or those of the publisher, the editors and the reviewers. Any product that may be evaluated in this article, or claim that may be made by its manufacturer, is not guaranteed or endorsed by the publisher.
